# Focused ultrasound of the caudal vena cava in dogs with cavitary effusions or congestive heart failure: A prospective, observational study

**DOI:** 10.1371/journal.pone.0252544

**Published:** 2021-05-28

**Authors:** Yen-Yu Chou, Jessica L. Ward, Lara Z. Barron, Shane D. Murphy, Melissa A. Tropf, Gregory R. Lisciandro, Lingnan Yuan, Jonathan P. Mochel, Teresa C. DeFrancesco

**Affiliations:** 1 Department of Veterinary Clinical Sciences, College of Veterinary Medicine, Iowa State University, Ames, Iowa, United States of America; 2 Department of Clinical Sciences, College of Veterinary Medicine, North Carolina State University, Raleigh, North Carolina, United States of America; 3 Hill Country Veterinary Specialists, Spicewood, Texas, United States of America; 4 Department of Veterinary Biomedical Sciences, College of Veterinary Medicine, Iowa State University, Ames, Iowa, United States of America; Universita degli Studi di Padova, ITALY

## Abstract

**Introduction:**

Ultrasonographic indices of the inferior vena cava are useful for predicting right heart filling pressures in people.

**Objectives:**

To determine whether ultrasonographic indices of caudal vena cava (CVC) differ between dogs with right-sided CHF (R-CHF), left-sided CHF (L-CHF), and noncardiac causes of cavitary effusion (NC).

**Materials and methods:**

113 dogs diagnosed with R-CHF (n = 51), L-CHF (30), or NC effusion (32) were enrolled. Seventeen of the R-CHF dogs had pericardial effusion and tamponade. Focused ultrasound was performed prospectively to obtain 2-dimensional and M-mode subxiphoid measures of CVC maximal and minimal size (CVC_max_ and CVC_min_), CVC_max_ indexed to aortic dimension (CVC:Ao), and CVC collapsibility index (CVC-CI). Variables were compared between study groups using Kruskal-Wallis and Dunn’s-Bonferroni testing, and receiver operating characteristics curves were used to assess sensitivity and specificity.

**Results:**

All sonographic CVC indices were significantly different between R-CHF and NC dogs (*P* < 0.001). Variables demonstrating the highest diagnostic accuracy for discriminating R-CHF versus NC were CVC-CI <33% in 2D (91% sensitive and 96% specific) and presence of hepatic venous distension (84% sensitive and 90% specific). L-CHF dogs had higher CVC:Ao and lower CVC-CI compared to NC dogs (*P* = 0.016 and *P* = 0.043 in 2D, respectively) but increased CVC-CI compared to the R-CHF group (*P* < 0.001).

**Conclusions:**

Ultrasonographic indices of CVC size and collapsibility differed between dogs with R-CHF compared to NC causes of cavitary effusions. Dogs with L-CHF have CVC measurements intermediate between R-CHF and NC dogs.

## Introduction

Ultrasonography of the inferior vena cava (IVC) is commonly utilized as a noninvasive estimate of right heart filling pressures in people [1–3]. Maximal diameter of and degree of inspiratory collapse of the IVC correlate well with catheter-based measurements of right atrial pressure [4, 5]. Current American Society of Echocardiography guidelines for sonographic evaluation of the right heart provide standardized protocols for IVC assessment, reference ranges for normal IVC measurements, and cutoffs for IVC measurements suggesting elevated right atrial pressure [6]. Clinical applications of IVC sonography include the bedside assessment of IVC collapsibility as a marker of intravascular volume status in critically ill patients [7–9] and diagnosis of right-sided congestive heart failure (R-CHF) [6, 10].

In veterinary medicine, previous studies have reported reference ranges for maximum caudal vena cava diameter (CVC_max_), CVC_max_ to aortic ratio (CVC_max_:Ao), CVC collapsibility index (CVC-CI), hepatic vein diameter, and gallbladder wall edema in healthy dogs [11–13]. These indices have been evaluated in dogs as potential markers of hypovolemia and systemic or pulmonary hypertension [14–18]. A small pilot study in 6 dogs with R-CHF showed that these dogs had CVC_max_ and CVC_min_ measurements above normal reference range [19], and another study reported that dogs with left-sided CHF (L-CHF) had larger and less collapsible CVC compared to dogs with earlier stages of degenerative mitral valve disease [20]. However, no previous veterinary studies have determined whether sonographic indices of the CVC are diagnostically useful to differentiate R-CHF (cavitary effusions associated with elevated central venous hydrostatic pressure, resulting from severe right heart disease) from dogs with cavitary effusion secondary to a noncardiac disease (such as neoplasia, decreased plasma oncotic pressure due to hypoalbuminemia, local or systemic inflammation, or ruptured viscera). Furthermore, no previous studies have compared sonographic indices of the CVC between dogs with intravascular volume overload associated with R-CHF versus L-CHF (pulmonary edema associated with elevated pulmonary venous hydrostatic pressure, resulting from severe left heart disease).

A potential clinical application of this imaging modality is the point-of-care identification or exclusion of R-CHF as a cause of canine cavitary effusions. Dogs presenting with cavitary effusions often have nonspecific findings on physical examination [21, 22]. Unless clinicopathologic analysis of the fluid reveals blood, urine, or septic exudate [23], final diagnosis for the etiology of cavitary effusion can be challenging [24] and typically requires advanced imaging (abdominal ultrasonography, echocardiography, and computed tomography), which require additional equipment and specialist expertise. A reliable point-of-care test to differentiate R-CHF from noncardiac (NC) causes of cavitary effusion, such as focused ultrasound, could help clinicians prioritize a patient’s subsequent diagnostic workup and determine indication for diuretic therapy.

The primary objective of this study was to determine whether focused ultrasonographic markers of right heart filling pressures, particularly CVC indices, differed between dogs with R-CHF compared to NC effusions or L-CHF. We hypothesized that focused ultrasonographic measures of the CVC would differ between disease groups, with R-CHF dogs having larger and less distensible CVC compared to NC dogs.

## Materials and methods

Procedures were approved by the Institutional Animal Care and Use Committees at Iowa State University (protocol number 1-18-8688) and North Carolina State University (protocol number 17-063-O). Informed owner consent was obtained for each patient. A power calculation determined that a population of at least 20 subjects in each group would be needed to detect a difference in sonographic markers of 10% with a statistical power of 0.90 and α = 0.05.

Client-owned dogs presented to the Iowa State University Lloyd Veterinary Medical Center and North Carolina State Veterinary Hospital were prospectively recruited for this study between October 27, 2018, and December 31, 2019. To be included in this study, dogs were required to have one or both of the following as assessed by the attending clinician: (1) cavitary effusion (peritoneal, pleural, or pericardial) of at least moderate volume identified sonographically (defined as fluid accumulation >2 cm such that the attending clinician deemed the volume sufficient for diagnostic centesis), or (2) cardiogenic pulmonary edema with clinical signs (increased respiratory rate or effort) warranting diuretic therapy. Exclusion criteria included lack of owner consent or instability of patient precluding focused ultrasound. Cases were also excluded if subsequent fluid analysis identified the effusion as hemorrhagic (packed cell volume >10%), septic exudate (neutrophilic inflammation with intracellular bacteria), or urine (fluid creatinine higher than serum creatinine).

The following data were collected from the patient’s physical examination on hospital presentation: patient signalment, body weight, temperature, heart rate, respiratory rate, presence and description of heart murmur, presence and description of arrhythmias, Doppler systolic blood pressure, and sedation protocol if applicable. Fluid location(s) (pulmonary edema, pleural effusion, ascites, pericardial effusion), presence or absence of cardiac tamponade, and centesis information (cavity for which centesis was performed, amount and characteristics of effusion obtained) were also recorded.

Focused ultrasound examinations were performed by ACVIM-boarded cardiologists or supervised cardiology residents using platform ultrasound units (EPIQ7, Philips Healthcare, Andover, Massachusetts) coupled to phased-array transducers (S5-1, S8-3, or S12-4; Philips Healthcare, Andover, Massachusetts). Dogs were stabilized prior to focused ultrasound (including centesis, sedation, oxygen supplementation, or diuretics) as deemed appropriate by the attending clinician. Images were obtained in all dogs according to a standard protocol as follows. Dogs were scanned in right lateral recumbency on a specialized echocardiography table with cutout allowing cardiac imaging from below. Starting at the right parasternal location, ultrasound cine-loops of the cardiac long-axis 4-chamber view and the short-axis view at the level of the LV were obtained using 2-dimensional (2D) echocardiography for measurement of the ratio of right ventricular to left ventricular dimension (RV:LV) in long-axis and short-axis [25]. Subxiphoid (subcostal) views were used to obtain 2D and M-mode images of the CVC in long-axis as it crossed the diaphragm [11, 14]. Images of the liver optimized for the hepatic veins and of the gallbladder in cross-section were also obtained. For cardiac images, cine-loops were recorded to include at least 5 cardiac cycles; for CVC images, 6-second cine-loops were obtained to capture the CVC during maximum inspiration and expiration. Acquisition of focused ultrasound images took approximately 30 seconds per site, for a total imaging time of less than 5 minutes.

Archived cine-loop and still images of sonographic right-sided cardiac markers were evaluated off-line by investigators blinded to patient identity. Five cardiac cycles in sinus rhythm for each measured cardiac parameter were averaged and used for further analysis. Maximal right ventricular end-diastolic dimension and maximal left ventricular end-diastolic dimension were obtained at the level of the right and left ventricular papillary muscles in long-axis 4-chamber view and short-axis 2-chamber view, respectively, and RV:LV was calculated in both long-axis and short-axis [25]. Ratio of the left atrial to aortic diameter was calculated from right parasternal short-axis measurements as previously described [26]. Maximum and minimum CVC diameters (CVC_max_ and CVC_min_) were measured in both 2D and M-mode by identifying the maximum and minimum visible diameters of the CVC [11, 14]. CVC_max_ was also indexed to aortic diameter from the right parasternal short-axis heart base view (CVC:Ao). Gallbladder wall thickness was measured from cross-sectional view from leading edge to leading edge. Presence of gallbladder wall edema was defined as a hypoechoic layer within the hyperechoic gallbladder wall [13, 27]. Presence of hepatic venous distension was noted by subjective recognition of distended hepatic veins with a “tree-trunk” appearance [12, 28, 29].

Final clinical diagnosis of the cause of cavitary effusions was determined by retrospective review of each patient’s entire medical record by a single independent investigator at each study location blinded to focused ultrasound results. The final diagnosis incorporated physical examination findings and diagnostic test results other than the above right-sided sonographic markers, including clinicopathologic and cytologic analysis of cavitary effusions, labwork (complete blood count, chemistry panel, urinalysis), advanced imaging (radiography, complete echocardiography [including 2-dimensional, M-mode, color and spectral Doppler, and tissue Doppler imaging], abdominal ultrasonography, computed tomography), and response to therapy. Dogs were assigned to one of three study groups based on the location and etiology of their cavitary effusions. The R-CHF group was defined as dogs with cavitary effusions and severe right heart disease identified on complete echocardiogram. Dogs with pericardial effusion and cardiac tamponade were included in the R-CHF group but also subanalyzed as separate group (PCEFF). The L-CHF group was defined as dogs with radiographic evidence of cardiogenic pulmonary edema and severe left heart disease identified on complete echocardiogram. Dogs with cavitary effusions of noncardiac etiology were categorized as the NC group. Dogs with severe cardiac disease who manifested both pulmonary edema and cavitary effusions (biventricular CHF) were categorized as R-CHF or L-CHF based on the predominant fluid location, considering clinical severity of each fluid accumulation and need for therapeutic centesis. Dyspneic dogs with pulmonary edema that also had trace to mild cavitary effusions not requiring centesis were categorized as L-CHF. Dogs requiring therapeutic abdominocentesis or thoracocentesis were classified as R-CHF, even if they also had mild pulmonary edema.

Statistical analyses were performed using commercial software (R software, version 3.5.1, R Foundation for Statistical Computing, Vienna Austria; IBM SPSS Statistics 25.0, IBM Corporation, Armonk, New York, USA). Normality of data was assessed using the Shapiro-Wilk test. Parametric data are reported as mean ± standard deviation, nonparametric data are reported as median (interquartile range), and categorical data are reported as count (percent). Outliers were included in all analyses; missing data were omitted. Variables were compared between study groups using Kruskal-Wallis rank-sum testing and post hoc pairwise Dunn’s testing with Bonferroni correction (two-tailed analyses with corrected *P*-values < 0.05 considered statistically significant). Univariate logistic regression was performed to evaluate clinical and ultrasound variables associated with diagnosis group. Final multivariable models were created with a forward inclusion threshold of alpha = 0.05 using a backwards stepwise procedure. Receiver operating characteristic curves were developed to assess diagnostic accuracy for prediction of diagnosis group, and sensitivity and specificity were calculated for each index.

## Results

The final study population of 113 dogs comprised 88 dogs from Iowa State University and 25 dogs from North Carolina State University (**S1 File**), and included 5 intact females, 52 spayed females, 14 intact males, and 42 castrated males. Various breeds were represented (46 breeds in total), with the most common breeds being mixed breed (n = 25), Yorkshire terrier (9), Cavalier King Charles spaniel (5), Chihuahua (5), German shepherd (5), golden retriever (4), and Labrador retriever (4). Other clinical variables describing the study population are shown in **Table 1.**

**Table 1:** Clinical data by study group.

Parameter	All dogs	R-CHF	L-CHF	NC
Number of dogs	113	51	30	32
Age (years)	9.8 (7.0–12.0)	9.3 (6.0–12.1)	10.6 (8.9–12.0)	8.3 (5.1–11.3)
Male, n (%)	56/113 (50)	27/51 (53)	14/30 (47)	15/32 (47)
Body weight (kg)	13.6 (7.0–27.2)	15.7 (7.7–26.6)	9.7 (4.9–18.8)	18.0 (7.5–29.8)
Rectal temperature (°C)	38.4 (37.9–38.8)	38.2 (37.8–38.7)	38.5 (38.1–38.7)	38.4 (38.2–38.8)
Heart rate (beats/minute)	140 (114–168)	140 (102–161)	**150 (130–180)**^**a**^	**128 (110–151)**^**a**^
Respiratory rate breaths/minute)	40 (30–52)	**36 (30–50)**^**a**^	**60 (44–76)**^**ab**^	**32 (28–44)**^**b**^
Murmur present, n (%)	67/113 (59)	**32 (63)**^**a**^	**28/30 (93)**^**ab**^	**7/32 (22)**^**b**^
Arrhythmia present, n (%)	29/113 (26)	14 (27)	10/30 (33)	5/32 (16)
Systolic blood pressure (mmHg)	123 (110–149)	137.5 (120–151.5)	**112 (106–128)**^**a**^	**138 (120–152)**^**a**^
Sedation, n (%)	24/113 (21)	15/51 (29)	3/30 (10)	6/32 (19)
Pulmonary edema, n (%)	33 (27%)	3 (5.9%)	30 (100%)	0 (0%)
Ascites, n (%)	73 (65%)	43 (84%)	6 (20%)	24 (75%)
Pleural effusion, n (%)	37 (33%)	14 (27%)	4 (13%)	19 (59%)
Pericardial effusion, n (%)	36 (32%)	26 (51%)	8 (27%)	2 (6%)

Study dogs (total n = 113) were diagnosed and grouped for analysis as either right-sided congestive heart failure (R-CHF), left-sided congestive heart failure (L-CHF), or noncardiac causes of cavitary effusion (NC). The R-CHF group included 17 dogs with pericardial effusion and tamponade (PCEFF) that were also analyzed as a separate subgroup (see Table 3). Continuous data are presented as median (interquartile range), and categorical data are presented as number and percentage of dogs with each finding. Bold font and superscript letters indicate significantly different values between study groups for that letter (*P* < 0.05 with Bonferroni correction). When multiple letters are listed, the value for the indicated group is significantly different from more than one other group. Location of fluid was not compared statistically between groups since fluid location contributed to the definition and classification of disease groups.

Of enrolled dogs, 51 (45%) dogs were categorized as having R-CHF, 30 (27%) as L-CHF, and 32 (28%) as NC causes of effusion. Seventeen dogs with R-CHF had pericardial effusion with tamponade (PCEFF subgroup), representing 33% of dogs with R-CHF and 15% of the total study sample. Underlying causes of R-CHF included pulmonary hypertension (n = 10), congenital heart diseases including pulmonic stenosis or tricuspid valve dysplasia (6), dilated cardiomyopathy (4), degenerative mitral valve disease (DMVD) or degenerative tricuspid valve disease (4), bradyarrhythmia (4), inflow obstruction due to compressive neoplasia (3), arrhythmogenic right ventricular cardiomyopathy (2), and iatrogenic circulatory overload (1). Causes of PCEFF were right atrial or auricular mass (n = 7), heart base tumor (3), idiopathic pericarditis (3), left atrial rupture (2), mesothelioma (1), and septic pericarditis (1). Causes of L-CHF were DMVD (n = 22), dilated cardiomyopathy (6), patent ductus arteriosus (1), and bradyarrhythmia (1).

Extracardiac neoplasia was the most common cause of NC effusion (n = 11), with lymphoma and hepatic masses being diagnosed most frequently (n = 3 each). Other diagnosed NC diseases included hypoalbuminemia (n = 9), chylothorax (3), pancreatitis (2), hepatopathy (2), and 1 dog with intra-abdominal cysts. Cause of effusion was ultimately unknown in 4 cases with incomplete diagnostic workup; these cases were assigned to the NC group based on complete echocardiography showing no evidence of structural cardiac disease.

No significant differences were detected between study groups in terms of age, sex, body weight, rectal temperature, presence of arrhythmia, or incidence of sedation (see **Table 1**). Heart rate differed between the L-CHF and NC group, with L-CHF dogs having higher heart rate compared to dogs with NC disease (*P* = 0.036). Respiratory rate was significantly higher in the L-CHF group compared to NC (*P* < 0.001) and R-CHF (*P* = 0.004) groups. Murmur incidence was higher in L-CHF dogs compared to NC (*P* < 0.0001) or R-CHF (*P* = 0.021) dogs, as well as in R-CHF dogs compared to NC (*P* = 0.0008). Systolic blood pressure was lower in L-CHF dogs compared to dogs with NC effusion (*P* = 0.047; see **Table 1**). Location of fluid accumulation (pulmonary edema, ascites, pleural or pericardial effusion) is also presented in **Table 1;** these data were not statistically compared between groups since fluid location contributed to the definition and classification of disease groups.

**Fig 1:**
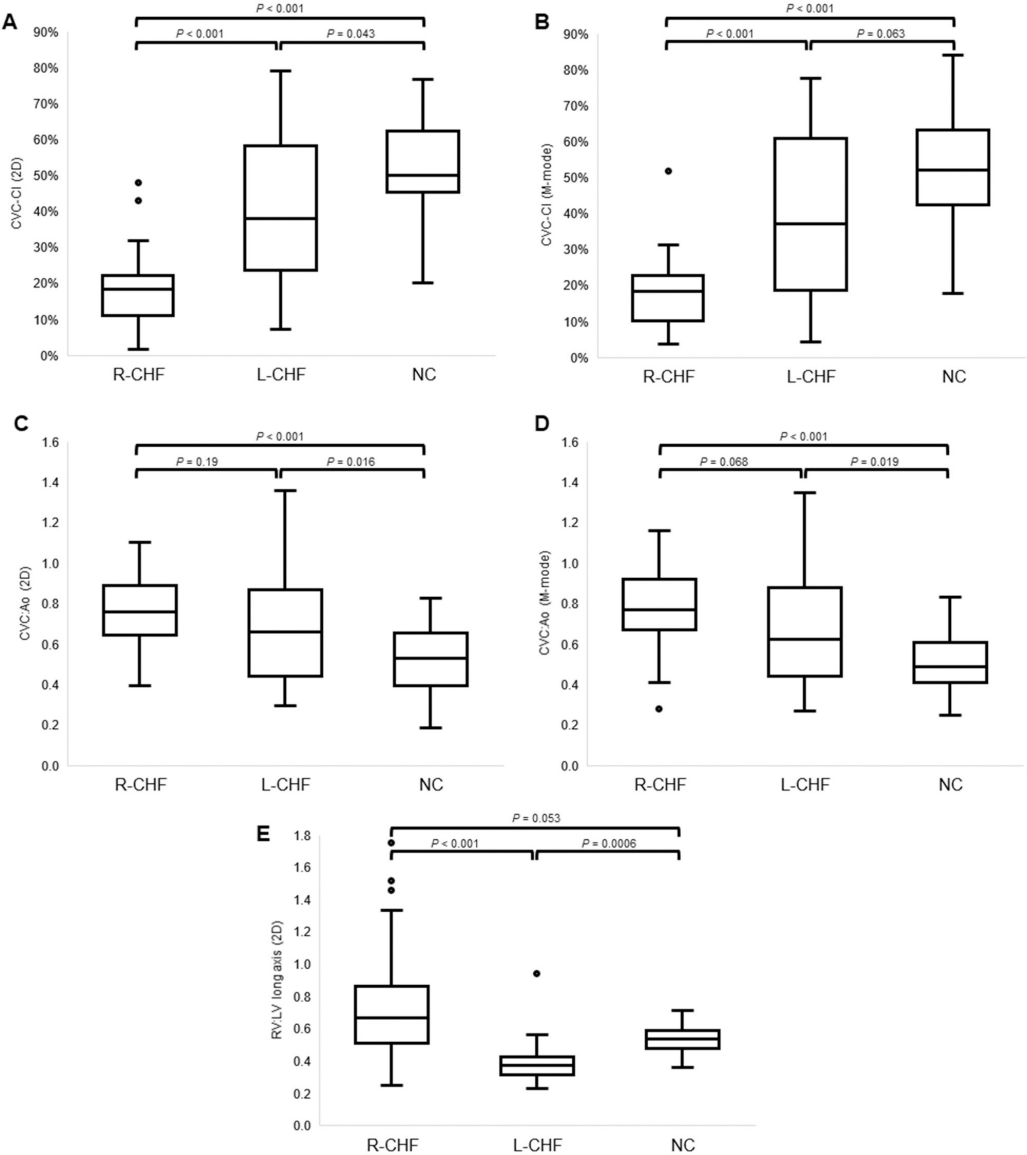
Selected ultrasound indices by study group. Box and whisker plots of data of select ultrasound indices obtained from 113 dogs with cavitary effusion or pulmonary edema diagnosed with either right-sided congestive heart failure (R-CHF, n = 51, including 17 dogs with pericardial effusion and tamponade), left-sided congestive heart failure (L-CHF, n = 30), or noncardiac causes of cavitary effusion (NC, n = 32). A, caudal vena cava collapsibility index (CVC-CI) in 2D; B, caudal vena cava collapsibility index in M-mode; C, maximum caudal vena cava to aorta ratio (CVC:Ao) in 2D; D, maximum caudal vena cava to aorta ratio (CVC:Ao) in M-mode; E, ratio of right ventricular to left ventricular dimension (RV:LV) in long axis. Boxes represent the interquartile range while the horizontal line in each box represents the group median; whiskers represent the 5th and 95th percentiles, and the outliers are plotted as dots.

**Fig 2:**
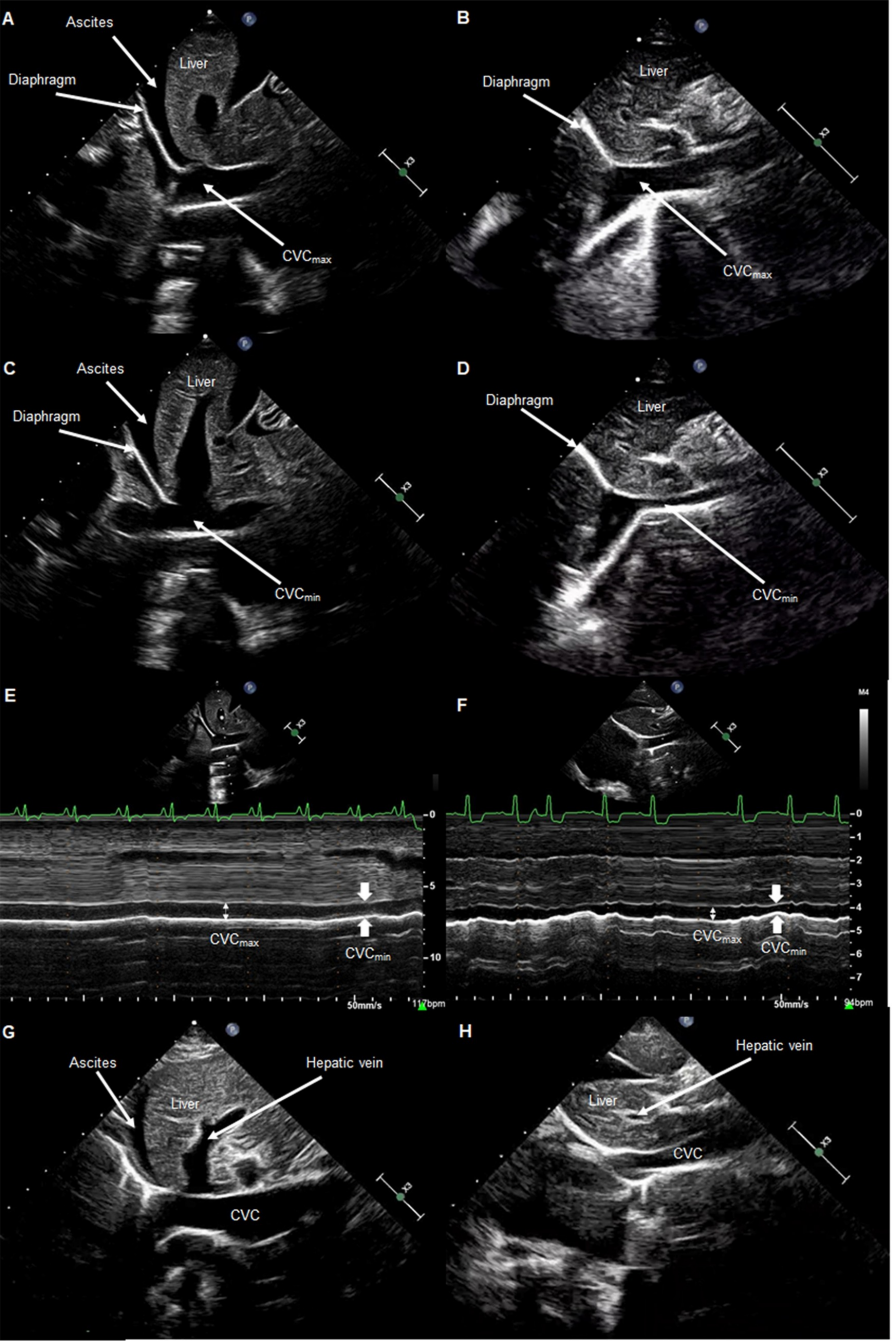
Representative focused ultrasound images. Representative images of caudal vena cava (CVC) sonographic indices taken from subxiphoid views in a patient with right-sided congestive heart failure (A, C, E, G) and a patient with noncardiac cause of cavitary effusion (B, D, F, H). A, B: Still 2-dimensional (2D) ultrasound images showing maximal sagittal diameter of the CVC (CVC_max_) as it crosses the diaphragm. C, D: Still 2D ultrasound images showing minimal sagittal diameter of the CVC (CVC_min_) as it crosses the diaphragm. E, F: M-mode image from a subxiphoid view showing CVC_max_ and CVC_min_ within a spontaneous respiratory cycle. G, H: Still 2D ultrasound images showing presence (G) and absence (H) of hepatic venous distension.

Performance of focused ultrasound was technically feasible in all dogs. Imaging was performed by a total of 2 cardiologists and 5 cardiology residents. Sonographic indices obtained from the three study groups are summarized in **Table 2.** Significant differences were found between groups for all target indices. Absolute CVC_max_ measurements were higher in dogs with R-CHF compared to NC or L-CHF dogs in both 2D (*P* = 0.0002 and *P* = 0.010, respectively) and M-mode (*P* = 0.0002 and *P* = 0.0004, respectively). CVC_min_ measurements were also higher in dogs with R-CHF compared to NC or L-CHF dogs in both 2D and M-mode (*P* < 0.0001 for all analyses). The CVC:Ao ratio was higher in the R-CHF and L-CHF groups compared to the NC group in both 2D (*P* < 0.001 and *P* = 0.016, respectively) and M-mode (*P* < 0.001 and *P* = 0.019, respectively). CVC-CI measurements in both 2D and M-mode were significantly lower in the R-CHF group compared to NC or L-CHF groups (*P* < 0.001 for all analyses). CVC-CI in 2D was also lower in the L-CHF group compared to the NC group (*P* = 0.043). The RV:LV ratio was lower in the L-CHF group compared to R-CHF and NC groups in both long-axis (*P* < 0.001 and *P* = 0.0006, respectively) and short-axis (*P* < 0.001 and *P* = 0.0026, respectively). Gallbladder wall thickness was higher in the R-CHF group compared to NC dogs (*P* = 0.0036), and hepatic venous distension was more common in the R-CHF group compared to either the L-CHF or NC groups (P < 0.001 for both analyses). **Fig 1** displays results for select sonographic indices by disease group. **Fig 2** provides example ultrasound images for CVC indices comparing representative dogs from the R-CHF versus NC groups.

**Table 2:** Focused ultrasound indices by study group.

Variable	R-CHF	L-CHF	NC
RV:LV (long axis, mm)	**0.67 (0.51–0.86)**^**a**^	**0.37 (0.32–0.42)**^**ab**^	**0.54 (0.48–0.59)**^**b**^
RV:LV (short axis, mm)	**0.53 (0.33–0.76)**^**a**^	**0.29 (0.23–0.39)**^**ab**^	**0.46 (0.37–0.52)**^**b**^
CVC_max_ (2D, mm)	**12.5 (10.7–15.9)**^**ab**^	**9.2 (6.3–13.8)**^**a**^	**8.9 (6.3–11.9)**^**b**^
CVC_min_ (2D, mm)	**10.1 (8.1–13.2)**^**ab**^	**4.5 (2.8–9.8)**^**a**^	**4.2 (2.7–6.3)**^**b**^
CVC_max_ (M-mode, mm)	**12.5 (10.9–16.2)**^**ab**^	**9.9 (5.8–13.0)**^**a**^	**9.3 (6.2–11.4)**^**b**^
CVC_min_ (M-mode, mm)	**10.7 (8.4–13.8)**^**ab**^	**4.2 (2.4–6.2)**^**a**^	**4.4 (2.4–6.3)**^**b**^
CVC-CI (2D, %)	**18.6 (11.4–22.3)**^**ab**^	**38.0 (25.0–55.7)**^**bc**^	**50 (45.4–60.7)**^**ac**^
CVC-CI (M-mode, %)	**18.3 (10.2–22.7)**^**ab**^	**37.2 (21.9–58.9)**^**bc**^	**52.0 (42.4–61.3)**^**ac**^
CVC:Ao (2D)	**0.76 (0.65–0.89)**^**a**^	**0.66 (0.45–0.84)**^**b**^	**0.53 (0.40–0.63)**^**ab**^
CVC:Ao (M-mode)	**0.77(0.67–0.92)**^**a**^	**0.63 (0.45–0.87)**^**b**^	**0.49 (0.43–0.61)**^**ab**^
Gallbladder wall thickness (mm)	**1.8 (1.3–2.2)**^**a**^	**1.1 (0.9–1.7)**^**b**^	1.6 (1.3–2.1)
Gallbladder wall edema (n, %)	7/49 (14.3)	2/30 (6.7)	2/32 (6.3)
Hepatic venous distension (n, %)	**45/50 (90)**^**ab**^	**12/30 (40)**^**a**^	**5/32 (15.6)**^**b**^

Study dogs (total n = 113) were diagnosed and grouped for analysis as either R-CHF (n = 51, 17 of which had PCEFF), L-CHF (n = 30), or NC causes of cavitary effusion (n = 41). Continuous data are presented as median (interquartile range), and categorical data are presented as number and percentage of dogs with each finding. Bold font and superscript letters indicate significantly different values between study groups for that letter (*P* < 0.05 with Bonferroni correction). When multiple letters are listed, the value for the indicated group is significantly different from more than one other group. 2D = 2-dimensional; CVC:Ao = ratio of maximal caudal vena cava diameter to aortic diameter; CVC-CI = caudal vena cava collapsibility index; CVC_max_ = maximal caudal vena cava diameter; CVC_min_ = minimal caudal vena cava diameter; RV:LV = ratio of right ventricular to left ventricular dimension.

Clinical and focused ultrasound variables were also subanalyzed within the R-CHF group, separating dogs with PCEFF from dogs with other causes of R-CHF (see **Table 3**). The only difference in clinical variables between these subgroups was lower incidence of heart murmur in PCEFF dogs (*P* = 0.0004), and the only difference in focused ultrasound variables was higher long-axis RV:LV ratio in dogs with other causes of R-CHF (*P* = 0.011; see **Table 3**).

**Table 3:** Clinical and focused ultrasound variables for dogs with right-sided congestive heart failure (R-CHF) subanalyzed by dogs with pericardial effusion and tamponade (PCEFF) versus other causes of R-CHF.

**Clinical variable**	**Other R-CHF**	**PCEFF**	**P-value**
Number of dogs	34	17	
Age (years)	9.1 (6.0–12.1)	9.8 (8.5–11.8)	1.00
Male, n (%)	18/34 (53)	9/17 (53)	1.00
Body weight (kg)	14.2 (7.1–23.2)	25.0 (12.9–32.8)	0.71
Rectal temperature (°C)	38.2 (37.8–38.7)	38.3 (37.9–38.6)	1.00
Heart rate (beats/minute)	140 (100–160)	130 (120–162)	1.00
Respiratory rate (breaths/minute)	36 (30–50)	40 (28–50)	1.00
Murmur present, n (%)	28/34 (82)	4/17 (24)	**0.0004**
Arrhythmia present, n (%)	12/34 (35)	2/17 (12)	0.43
Systolic blood pressure (mmHg)	121 (113–148)	130 (119–145)	1.00
Sedation, n (%)	11/34 (32)	4/17 (24)	1.00
Pulmonary edema, n (%)	3 (9%)	0 (0%)	--
Ascites, n (%)	34 (100%)	9 (53%)	--
Pleural effusion, n (%)	5 (15%)	9 (53%)	--
Pericardial effusion, n (%)	9 (27%)	17 (100%)	--
**Focused ultrasound variable**	**Other R-CHF**	**PCEFF**	**P-value**
RV:LV (long axis, mm)	0.79 (0.61–0.93)	0.55 (0.47–0.65)	**0.023**
RV:LV (short axis, mm)	0.67 (0.36–074)	0.45 (0.29–0.41)	0.060
CVC_max_ (2D, mm)	12.1 (10.6–15.1)	13.0 (10.8–17.8)	1.00
CVC_min_ (2D, mm)	9.9 (8.3–12.9)	12.0 (7.7–13.6)	1.00
CVC_max_ (M-mode, mm)	12.1 (10.9–15.3)	13.5 (11.3–16.9)	1.00
CVC_min_ (M-mode, mm)	10.6 (8.5–13.4)	10.8 (8.0–14.0)	1.00
CVC-CI (2D, %)	16.8 (10.9–20.9)	20.5 (17.5–23.6)	1.00
CVC-CI (M-mode, %)	17.1 (9.6–20.0)	21.5 (17.4–26.4)	1.00
CVC:Ao (2D)	0.76 (0.67–0.90)	0.68 (0.60–0.85)	1.00
CVC:Ao (M-mode)	0.77 (0.69–0.94)	0.75 (0.65–0.83)	1.00
Gallbladder wall thickness (mm)	1.7 (1.3–2.1)	1.8 (1.3–2.2)	1.00
Gallbladder wall edema (n, %)	3/32 (9%)	4/17 (24%)	0.85
Hepatic venous distension (n, %)	33/34 (97%)	12/16 (75%)	0.87

Continuous data are presented as median (interquartile range), and categorical data are presented as number and percentage of dogs with each finding. Significant differences between groups are indicated in bold (*P* < 0.05 with Bonferroni correction). See Table 2 legend for explanation of abbreviations.

Receiver operating characteristic curves were performed to identify clinical and sonographic indices that were predictive of different disease groups. Variables found to be independently predictive in multivariate regression are listed in **Table 4**. **Table 5** depicts sensitivity and specificity of bodyweight-independent focused sonographic indices for the differentiation of R-CHF (including PCEFF) versus NC causes of effusion, representing the clinical scenario wherein focused ultrasound may provide the most useful diagnostically relevant information.

**Table 4:** Clinical and sonographic variables useful for predicting disease group.

Variable		Threshold value	AUC	95% CI	Sensitivity (%)	Specificity (%)	P-value
*R-CHF (including PCEFF) versus all other dogs; threshold to predict R-CHF*							
	RV:LV Lax	>0.55	0.79	0.70–0.88	66.7	72.6	0.0072
	CVC-CI (2D, %)	<26.2	0.89	0.83–0.96	88.2	82.3	<0.0001
*R-CHF (including PCEFF) versus NC; threshold to predict R-CHF*							
	CVC-CI (2D, %)	< 33.4	0.97	0.94–1.00	90.6	96.1	0.0231
	Hepatic venous distension	Present			84.4	90	0.0255
*R-CHF (including PCEFF) versus L-CHF; threshold to predict R-CHF*							
	CVC-CI (2D, %)	< 26.3	0.80	0.69–0.92	88.2	70	0.0011
*PCEFF versus other causes of R-CHF; threshold to predict PCEFF*							
	RV:LV Lax	< 0.72	0.80	0.68–0.92	94.1	61.8	0.0120
	Murmur	Absent			76.5	82.4	0.0014

Listed variables were significantly predictive of disease group using multivariate modeling of receiver operating characteristic curves. Study sample included 113 dogs diagnosed with R-CHF (n = 51, 17 of which had PCEFF), L-CHF (n = 30), or NC causes of cavitary effusion (n = 32). No variables were found to be predictive of L-CHF versus NC effusion in multivariate analysis. See Table 2 legend for explanation of abbreviations.

**Table 5:** Diagnostic accuracy of bodyweight-independent focused ultrasound variables to differentiate R-CHF (including PCEFF) from NC causes of cavitary effusion.

Variable	Threshold	Sensitivity	Specificity
CVC-CI (2D, %)	< 33%	90.6%	96.1%
CVC-CI (M-mode, %)	< 29%	90.6%	92.1%
CVC:Ao (2D)	> 0.62	74.2%	80.4%
CVC:Ao (M-mode)	> 0.63	80.6%	80.4%
RV:LV (long axis, mm)	> 0.64	90.6%	56.9%
RV:LV (short axis, mm)	> 0.54	81.3%	47.1%
Hepatic venous distension	Present	84.4%	90.0%
Heart murmur	Present	78.1%	62.7%

Threshold values indicate cutoff to identify R-CHF. See Table 2 legend for explanation of abbreviations.

## Discussion

As hypothesized, the focused ultrasound indices investigated in this study differed between disease groups and were diagnostically useful for prediction of R-CHF as a cause of cavitary effusions in dogs. Variables with the highest sensitivity and specificity for detection of R-CHF included CVC-CI, CVC:Ao, and presence of hepatic venous distension. These findings are logical since elevated central venous hydrostatic pressure in patients with R-CHF is transmitted to the CVC, resulting in increased size and decreased distensibility of this vessel and the hepatic veins.

This study represents the first investigation of focused ultrasound parameters for the diagnosis of R-CHF in veterinary medicine. Diagnostic accuracy of CVC-CI reported in our study (sensitivity 91% in both 2D and M-mode; specificity 96% in 2D, 92% in M-mode) was similar or higher than inferior vena cava collapsibility index (IVC-CI) accuracy in multiple human studies [8, 9]. In people, IVC-CI with a cutoff of <22% was 78% sensitive and 98% specific for differentiating patients with R-CHF from healthy patients without cardiac or lung disease [10]. In another study, IVC-CI with a cutoff of <15% was 93% sensitive and 84% specific for a diagnosis of CHF versus NC causes of respiratory distress [30], and IVC-CI with a cutoff of <20% was 52% sensitive and 86% specific for differentiating patients with acute decompensated CHF from those with noncardiac causes of acute dyspnea [31]. In elderly patients with dyspnea, IVC-CI with a cutoff of <50.5% was 84% sensitive and 91% specific for detecting CHF versus primary pulmonary disease [32].

Predictive performances of focused ultrasound for detection of R-CHF versus NC disease in our analysis was higher than in a previous study investigating the utility of similar sonographic indices to predict pulmonary hypertension in dogs, which reported only 11% sensitivity and 88% specificity [14]. These variations can be explained by differences in study design and inclusion criteria. In the prior study, dogs with mild to severe pulmonary hypertension were included; while these dogs by definition had elevated right ventricular *systolic* pressure, few would be expected to have elevated right ventricular *diastolic* (right atrial) pressure. Changes to CVC size or distensibility would only be expected in dogs with advanced right heart disease leading to elevated central venous pressure and R-CHF. In our study, only dogs diagnosed with overt R-CHF (cavitary effusion) secondary to their right heart disease were included in the R-CHF group.

In addition to differentiating R-CHF and NC effusions, several parameters evaluated in this study also performed well in discriminating other disease groups. Indices that best differentiated L-CHF from other groups included respiratory rate and RV:LV ratio in long-axis, explained by the enlargement of left-sided cardiac chambers and presence of pulmonary edema in dogs with L-CHF. The L-CHF group was also distinguishable from the NC group using CVC-CI, CVC:Ao, and hepatic venous distension; L-CHF dogs had values for these parameters that were clinically and statistically intermediate between the R-CHF and NC groups. This is consistent with findings from a previous study in dogs with DMVD, which reported higher CVC:Ao and decreased CVC:CI in dogs with stage C or D (previous or current L-CHF) compared to dogs in earlier stages of disease [20]. These results suggest that the increased intravascular volume in dogs with L-CHF does lead to hypervolemia and demonstrably elevated central venous hydrostatic pressure, though not to the same degree as in R-CHF.

Dogs with PCEFF and cardiac tamponade were included in the R-CHF group in this study because the pathophysiology of other cavitary effusions in cardiac tamponade (elevated central venous hydrostatic pressure) is the same as other causes of R-CHF; therefore, similar changes to the CVC and hepatic vasculature would be expected. However, because PCEFF represents a distinct clinical scenario (obstruction to right heart filling rather than right heart pressure or volume overload), subanalyses were also performed comparing dogs with PCEFF to other causes of R-CHF. Not surprisingly, the only variables that differed between these subgroups were incidence of heart murmur and RV:LV ratio. This reflects the fact that dogs with PCEFF and tamponade typically have structurally normal hearts, while dogs with other causes of R-CHF have severe right heart remodeling.

Interestingly, results of this study revealed a much larger spread of CVC-CI measurements for the L-CHF group compared to other disease groups. We speculate that this disparity occurred because the change in the size of the CVC depends on the change in intrathoracic pressure during respiration. Respiratory rate and degree of respiratory distress were higher and more variable within the L-CHF group compared to the other disease groups, wherein most dogs had normal respiratory rate and effort. This variation in respiratory effort and thus intrathoracic pressure during respiration in dyspneic dogs could cause more variability in magnitude of CVC collapse, or more translational motion affecting ability to image the center of the CVC. The influence of diaphragmatic movement on IVC-CI has been demonstrated in humans [33, 34]. However, this would be challenging to confirm as it is difficult to quantify magnitude of respiratory effort in spontaneously breathing dogs.

There was no statistically significant difference in gallbladder wall edema between the study groups, suggesting that presence or absence of gallbladder wall edema is a poor screening test for R-CHF (or PCEFF specifically). This is not necessarily surprising, as a wide variety of noncardiac causes for gallbladder wall edema have been identified, including anaphylaxis [35], hepatitis, hypoproteinemia [36], volume overload [12], and cholecystitis [37]. Interestingly, RV:LV in short axis had relatively low sensitivity and specificity (81% and 47%, respectively) for differentiating the R-CHF and NC groups, while the same ratio in long-axis performed better (91% sensitivity and 57% specificity). This may reflect the crescent-shaped geometry of the right ventricle in short axis as well as more variability in this scanning plane, which can lead to underestimation of chamber size in this view, particularly in dogs with concurrent left heart disease.

Among dogs with R-CHF (but *excluding* dogs with PCEFF and cardiac tamponade), ascites was present in all 34 (100%) dogs, trace PCEFF was present in 9 (27%) dogs, and pleural effusion was only present in 5/34 (15%) cases; isolated pleural effusion occurred exclusively in the NC group. In a previous study of dogs with right-sided manifestations of CHF secondary to dilated cardiomyopathy or DMVD, 40/60 (67%) of dogs with R-CHF had ascites, 26/60 (43%) had pleural effusion, 13/60 (22%) had PCEFF, and similarly no dogs had pleural effusion only [38]. The higher incidence of ascites and lower incidence of pleural effusion in the present study might reflect the difference in inclusion criteria between studies, since the prior study described dogs with primarily left-sided heart disease who also had cavitary effusions as part of their manifestation of CHF. Together, these studies suggest that absence of ascites, and particularly the finding of isolated pleural effusion, should significantly decrease clinical suspicion for R-CHF.

One of the limitations of this study was that no healthy control group was recruited for comparison of CVC indices. Measurements of 2D CVC_max_ and CVC_min_ from the subxiphoid view have been previously reported in healthy dogs [11, 39], and bodyweight-normalized reference intervals for canine CVC diameter have been published from the paralumbar and hepatic views [11, 12]. Absolute values for CVC_max_ in the NC group of the present study (9.3 +/- 6.0 mm) were similar to measurements reported from the subxiphoid view in similarly-sized healthy dogs (8.7 +/- 2.7 mm [11]; 8.0–12.0 mm [39]). The majority of CVC measurements from our NC group (76% for 2D, 85% for M-mode) were also within the reported bodyweight-normalized 95% reference interval for healthy dogs from the paralumbar view [11]. Thus although the present study did not specifically recruit a cohort of control dogs, our results suggest that dogs with NC causes of cavitary effusion generally have CVC size and distensibility comparable to that of normal dogs, while dogs with R-CHF and L-CHF have vena cavae that are larger and less distensible.

Our study had several additional limitations. Investigators were unlikely to enroll unstable dogs or those requiring emergency surgery. Time between hospital presentation and focused ultrasound was not standardized, although the examination typically occurred within 12 hours of presentation; it is possible that the disease status could have changed during that time period, particularly if treatment for CHF (e.g. furosemide) or intravenous fluids had been administered before ultrasound was performed. Operators were not blinded to the presumptive diagnosis of the patient at the time of focused ultrasound, which may have led to bias during image acquisition. It is possible that sedative drugs may have affected central venous pressure (and thus CVC measurements) in some dogs, although use of sedation did not differ between disease groups. Additionally, a minority of dogs with CHF had evidence of both pulmonary edema and cavitary effusions, and this pathophysiologic overlap may have obscured some differences between R-CHF and L-CHF groups.

A further limitation is that focused ultrasound examinations in this study were performed by cardiologists or cardiology residents utilizing a platform cardiac ultrasound machine and specialized echocardiography table. Personnel and equipment would be different in a primary care setting or emergency department, which is the intended setting for application of these techniques as a focused ultrasonographic screening tool. Differences in the operators, ultrasound model, probes, and software can lead to disparities in imaging quality and results; future studies may assess diagnostic accuracy of these measurements performed by non-specialists in a point-of-care setting. Additionally, interobserver reliability was not assessed in this study. Previous studies show good to excellent inter-rater agreement of CVC measurements performed at the hepatic, paralumbar, and spleno-renal views [11, 16, 18]. A study of ultrasound measurement at subxiphoid view in 15 healthy Beagle dogs reported that interobserver agreement between non-cardiologists and cardiologists was acceptable for CVC_max_, but not for CVC_min_ or CVC-CI [40]. Therefore, our results obtained from the subxiphoid view may have been different from those of other operators or observers. This is important because CVC measurements taken from planes that do not transect the actual middle of the vessel can underestimate the true diameter, and this can occur during the shift of CVC during inspiration and expiration. We chose to perform ultrasound of the CVC at the subxiphoid view using the sagittal imaging plane based on the authors’ clinical experiences suggesting that this view is the easiest to obtain reliably and is the currently the most commonly performed in practice; additionally, this view and orientation are most similar to the preferred protocol for imaging the IVC in people [6]. As with all focused ultrasound examinations, CVC findings must be integrated with other clinical and imaging findings when making emergent diagnostic and treatment decisions.

In conclusion, the present study demonstrated that focused ultrasound of the CVC is a feasible and accurate diagnostic test for the detection of R-CHF in dogs with cavitary effusion. Dogs with NC effusion had CVC size and collapsibility similar to normal dogs, while dogs with R-CHF had large non-distensible CVC and hepatic venous distension, and dogs with L-CHF had CVC measurements in between these extremes. CVC-CI in either 2D or M-mode was the best ultrasonographic index to differentiate between study groups, and a cutoff value of less than approximately 30% was more than 90% sensitive and specific for diagnosis of R-CHF versus NC effusion. Focused ultrasound of the CVC could have diagnostic utility to increase or decrease index of suspicion for R-CHF as the cause of cavitary effusions.

## Supporting information

S1 File Clinical and focused ultrasonographic data for the full study population (n = 113 dogs). (XLSX)
